# Drawing the borders of the mesophotic zone of the Mediterranean Sea using satellite data

**DOI:** 10.1038/s41598-022-09413-4

**Published:** 2022-04-04

**Authors:** Giorgio Castellan, L. Angeletti, P. Montagna, M. Taviani

**Affiliations:** 1 Institute of Marine Sciences, National Research Council (CNR-ISMAR), Via Gobetti 101, 40129 Bologna, Italy; 2 Department for the Cultural Heritage, University of Bologna, Via degli Ariani 1, 48121 Ravenna, Italy; 3 Institute of Polar Sciences, National Research Council (CNR-ISP), Via Gobetti 101, 40129 Bologna, Italy; 4 Lamont Doherty Earth Observatory of Columbia University, 61 Route 9W, Palisades, NY 10964 USA; 5 Biology Department, Woods Hole Oceanographic Institution, 266 Woods Hole Road, Woods Hole, MA 02543 USA; 6 Stazione Zoologica Anton Dohrn, Villa Comunale, 80121 Naples, Italy

**Keywords:** Ecology, Environmental sciences

## Abstract

The 30–150 m bathymetric range is commonly adopted in the literature to constrain the mesophotic zone. However, such depth interval varies depending on sunlight penetration, which is primarily a function of solar radiation incidence and water clarity. This is especially obvious in the Mediterranean Sea with its peculiar biophysical properties. Integrating information on light regime in the estimation of the bathymetric range of the mesophotic zone would provide a more robust definition, orienting conservation actions targeting its ecosystems. We present a first assessment of the spatial and vertical extension of the mesophotic zone in the Mediterranean Sea based upon light penetration, comparing our prediction with literature data. Our study also represents a baseline to monitor future variations in the bathymetric interval associated with the mesophotic zone in the Mediterranean Sea in relation to global changes.

## Introduction

Marine ecosystems are currently facing multiple stressors from human-induced and natural pressures^1–3^. The definition and rectification of management and conservation goals together with building effective governance are arguably amongst the best actions for the future survival of marine ecosystems^4^. To do this appropriately and effectively, not only a sound scientific comprehension of the dynamics governing marine ecosystems is required, but also a greater knowledge of their spatial distribution and extension is needed to orient future research and inform management plans and conservation measures^5–7^.

Setting the borders to properly design conservation actions may be challenging since natural ecosystems distribute along environmental gradients and may lack obvious boundaries. This is the case of the mesophotic zone that occupies the lower portion of the euphotic zone, where irradiances sustain net positive photosynthesis, whose lower bound (i.e., compensation point) is historically set at 1% of the surface Photosynthetically Active Radiation (PAR^8^).

Although long since recognized^9,10^, the mesophotic zone got comparatively less attention by the scientific community in favor of the shallower and deeper counterparts. A reverse is taking place in the last decades, with an exponential increase of the volume of scientific literature on mesophotic zone^11^ that provided a wealth of information on the ecological importance of mesophotic ecosystems, including their ability to provide a refuge for shallow-water species as well as areas for spawning, breeding, feeding, and growth to maturity^8,12–14^. This has been favored by dedicated projects and funds from public agencies/organizations and private foundations to directly support the exploration of ecosystems populating the mesophotic zone (e.g., NCCOS, UNEP, Schmidt Ocean Institute, Oceana^15^).

In terms of bathymetric range, the most agreed definition of the mesophotic zone sets the limits from 30–40 m to ca. 150 m water depth in tropical and subtropical regions^8^. Pragmatically, the upper boundary of the mesophotic zone originates from physiologically imposed depth limits of conventional SCUBA diving that largely restricted the exploration to 30–40 m, which also represent the deeper portions of shallow-water zooxanthellate coral communities^9,16–19^. This boundary also reflects the depths where a shift from photophilous (thriving in full light) to sciaphilous (tolerant to shade) organisms usually occurs^12^. The lower boundary at ca. 150 m water depth derives from the deepest occurrences of zooxanthellate corals documented in the late 80s (e.g., in the Caribbean at 119 m^17^, in the Red Sea at 145 m^20^, in Hawaii at 153 m^21^, and in Johnston Atoll at 165 m^22^).

The consistency of such depth limits for tropical and sub-tropical situations has been verified by exploring patterns of distribution and composition of mesophotic coral ecosystems (MCEs) that provided evidence of a subdivision in an upper (30–60 m) and a lower mesophotic zone (60–150 m), which host communities different in composition^8,23–26^. The depth range associated with mesophotic conditions (30–150 m), and the community break at ~ 60 m depth were proved to be robust also through meta-analysis considering information on benthic tropical mesophotic reefs from around the world^27^, and studies based on irradiances (^28–30^ among others).

Nevertheless, situations dominated by zooxanthellate scleractinian corals represent a minority in the benthic assemblages characterizing the mesophotic depths at the global scale^11,29^. At the temperate latitudes, observations of light-dependent corals appear to be sparse and mesophotic ecosystems are typically dominated by octocorals, mainly gorgonians and antipatharians and sponges (e.g.,^31–34^), but include also bryozoans, ascidians, and shade-adapted (sciaphilous) algae^14^. The strong seasonal variability that characterizes temperate systems influences the water clarity affecting the penetration of solar radiation along the water column (e.g.,^35^). Hence, the interval 30–150 m might not necessarily represent the bathymetric range of mesophotic condition occurrence at these latitudes.

The Mediterranean Sea, for instance, is a mid-latitude semi-enclosed and oligotrophic basin, with complex oceanographic dynamics, strong seasonality^36,37^, and peculiar physico-chemical properties, such as high salinity, temperature and density^38–40^. Strong climatic (e.g., rainfalls, sunlight), oceanographic (e.g., water temperature and salinity) and bio-geochemical (e.g., nutrients) gradients characterize the basin^41–43^, generating an alternation of temperate- and tropical-like conditions within a relatively short distance (about 4000 km from the Strait of Gibraltar to the Gulf of Iskenderun, southeastern coast of Turkey). This environmental complexity controls the structure and composition of benthic communities in the Mediterranean Sea. Zooxanthellate corals thrive mainly at depths shallower than 30 m (e.g.,^44^), with mesophotic ecosystems represented by a mosaic of assemblages composed of algae, cnidarians, sponges, bryozoans, crinoids, brachiopods, and ascidians, both on hard and soft substrates (e.g.,^32–34,45–56^ among others).

However, information on Mediterranean mesophotic ecosystems (including studies using the term “twilight” to refer to mesophotic situations) is limited to few taxonomic groups (mainly cnidarians and sponges^31^) while other assemblages have been explored only seldom (e.g., brachiopod-dominated communities and deep-sea oyster reefs^49,50^). The low number of studies of Mediterranean mesophotic ecosystems presenting taxonomic components other than cnidarians^31^ might not be a consequence of the lower interest from the scientific community. Rather, the term “mesophotic” is likely less used to define situations without a coral component. Among these are rhodolith beds and coralligenous habitats, studied widely in the Mediterranean Sea^57,58^. If the former are increasingly accounted as mesophotic (e.g.,^58,59^), opinions differ on whether coralligenous assemblages should be considered an independent biological feature or under the mesophotic domain (e.g.,^31^). In terms of light regime, these situations might be included in the mesophotic zone since they occur in sciaphilous conditions and at mesophotic depths^60,61^.

The lack of a clear definition of the borders of the mesophotic zone at temperate latitudes may not pose much of a problem as long as the term “mesophotic” intends to refer to intermediate-depth biological situations. Problems arise when scientific information must be transferred to policies that require a coherent spatial definition to plan proper management and conservation.

The mesophotic zone extends from 30 m to where photosynthesis compensation point occurs. This lower boundary is, however, variable due to differences in light penetration, and environmental and ecological drivers that can vary over short distances^12,62^. Observations of live macroalgae assemblages, mainly coralline algae, that are able to maintain active photosynthesis at irradiance levels as low as 0.0001 mol photons m^−2^ day^−1^ (corresponding to 0.0005% of surface PAR,^63,64^), suggest that the photic zone could extend deeper than previously assumed.

If we were able to include the light regime in the definition of the mesophotic zone at temperate latitudes, we could not only constrain its bathymetric range and estimate the portion of seafloor characterized by mesophotic conditions but also appreciate variations related to local factors. Drawing the limits of the temperate mesophotic zone under current climatic conditions would be very important especially in the context of future global changes. According to IPCC predictions, nutrients supply and ocean productivity will likely decline in the near future, largely affecting phytoplankton communities and the transparency of the water column^65^. On the other hand, changes in precipitation, storm surges, and heatwaves related to ongoing climatic variations have already increased the volume of sediment loads from river runoff, affecting the turbidity of the water column^66–68^. Consequently, the vertical extension of the photic and mesophotic zones will change, affecting the ecosystem structure of these environments with taxonomic groups that might take advantage in the long term of a concomitant decline of more sensitive organisms^65,69,70^.

In the Mediterranean Sea, mesophotic communities are already threatened by natural and anthropogenic pressures, including seawater temperature increase, heat waves, bottom trawling and marine litter (e.g., abandoned or derelict fishing gear), which trigger a gradual but irreversible process of habitat degradation (e.g.,^71–73^). A better understanding of the bathymetric and spatial extension of mesophotic zone in the Mediterranean Sea is, thus, urgent to orient conservation actions. However, the penetration of PAR along the water column depends on complex dynamics involving the inherent optical properties (IOPs) of the water column, the combined effects of absorption and scattering, and the amount and on the directionality of the light at the surface, despite the latter has been proven to be weak away from boundary effects, such as at mesophotic depths^74^. For such a reason, identifying the borders of the mesophotic zone that rely upon light regime might be challenging when on-site irradiance and IOPs measurements are not available.

Here we present a first assessment of the seabed portion ascribable to mesophotic conditions for the entire Mediterranean Sea based on irradiance penetration derived from satellite data. Our estimation is then validated with literature data on benthic mesophotic assemblages. By providing its current spatial and bathymetric extension, we provide a baseline to monitor the future variation of the mesophotic zone of the Mediterranean Sea in response to global changes.

## Results

### Extension of the mesophotic zone

**Figure 1:**
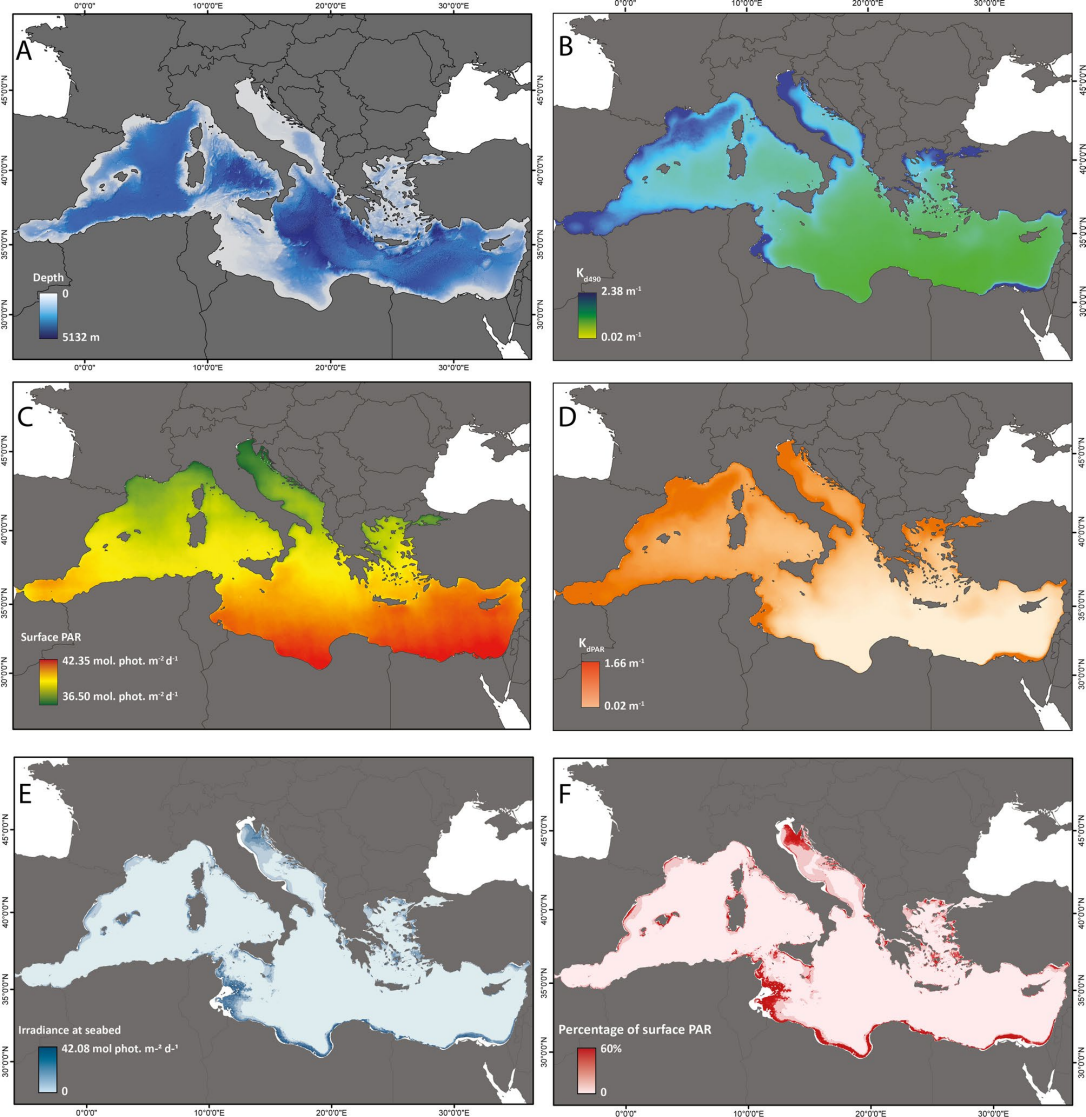
Bathymetry, satellite data and estimated light at seabed. (**A**) Bathymetric data for the Mediterranean Sea; (**B**) diffuse attenuation coefficient at 490 nm from satellite data in the Mediterranean Sea (K_d490_); (**C**) Surface Photosynthetically Active Radiation (PAR); (**D**) Diffuse attenuation coefficient for PAR light (K_dPAR_) calculated from K_d490_, according to method proposed by^59^ and^66^; (**E**) Quantity and (**F**) Fraction of surface PAR reaching the seabed for the Mediterranean Sea. Data were sourced from the NASA Ocean Color database (https://oceancolor.gsfc.nasa.gov/). Maps were generated using ArcMap 10.5 software (© ESRI, https://desktop.arcgis.com).

**Figure 2:**
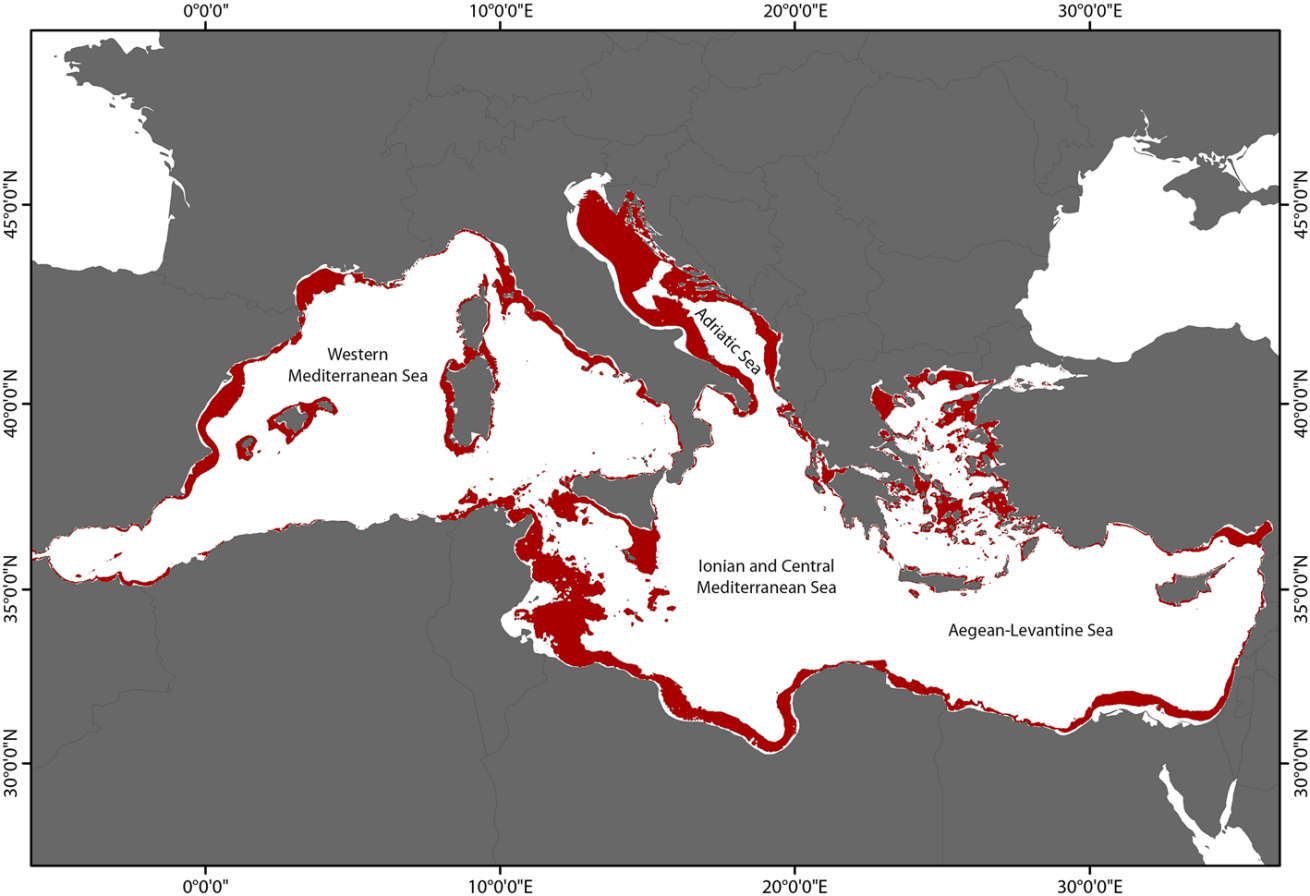
Estimated spatial extent of the mesophotic zone in the Mediterranean Sea. Portions of seabed characterized by mesophotic conditions (red shaded area). The upper and lower limits were set at 30 m depth and at 0.0001 mol photons m^−2^ day^−1^ of irradiance, respectively. Map generated using ArcMap 10.5 software (© ESRI, https://desktop.arcgis.com).

**Figure 3:**
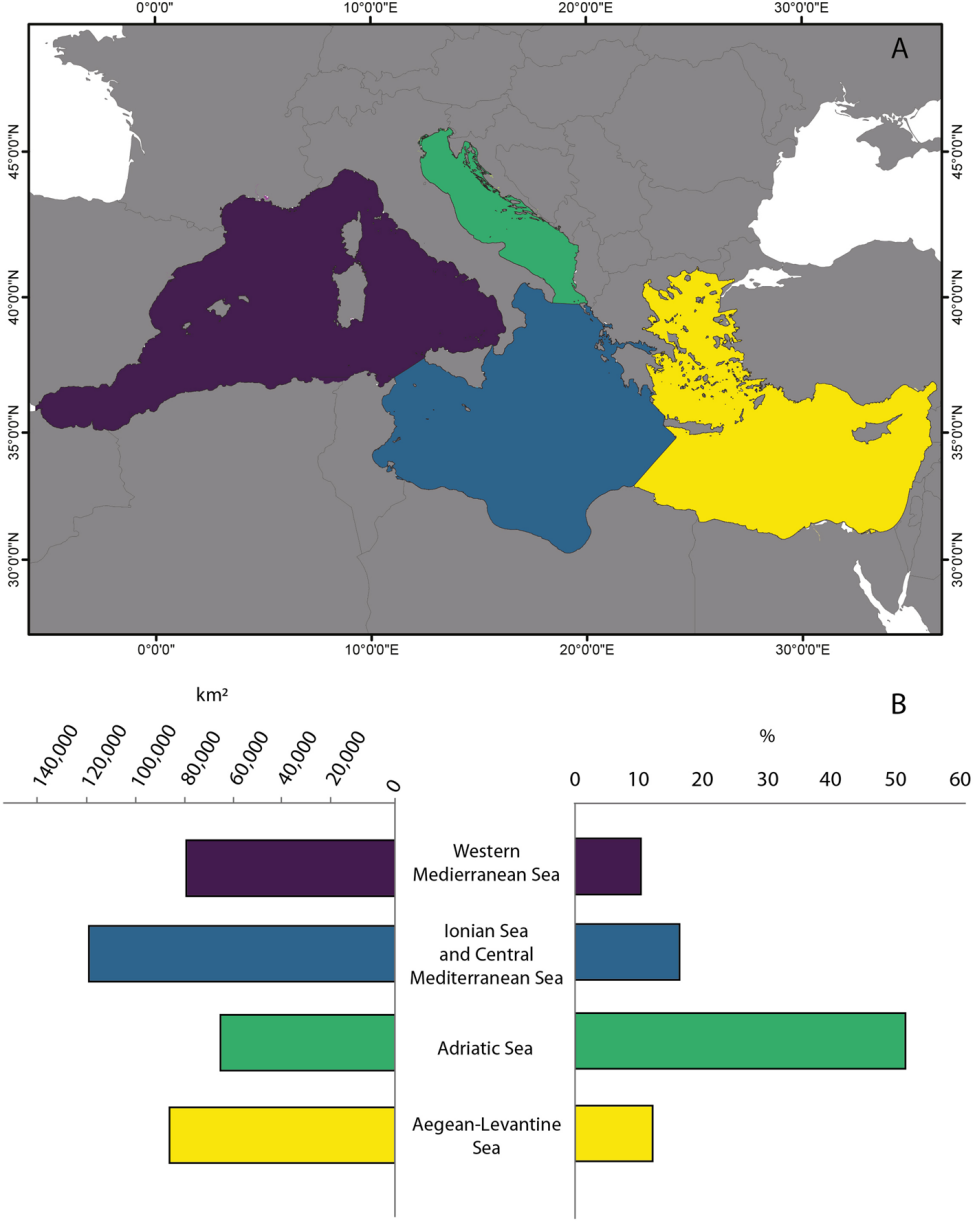
Portion of seabed under mesophotic conditions among Mediterranean sub-regions. (**A**) Sub-regions of the Mediterranean Sea according to the MSFD (2008/56/EC); Western Mediterranean Sea (purple): ca. 844,417 km^2^; Ionian and Central Mediterranean Sea (blue): 772,705 km^2^; Adriatic Sea (green): 139,300 km^2^; Aegean-Levantine Sea (yellow): 756,642 km^2^. Map generated using ArcMap 10.5 software (© ESRI, https://desktop.arcgis.com). (**B**) Bar plot showing the portion of seabed under mesophotic conditions in the different MSFD sub-regions in terms of km^2^ and areal percentage.

The portion of seabed characterized by mesophotic conditions varies in its extension across the Mediterranean Sea in relation to the latitude and the longitude (Figs. 1, 2). Our study considers the four main sub-regions of the Mediterranean Sea (Fig. 3A) according to the Marine Strategy Framework Directive (MSFD; 2008/56/EC). We observed a NNW–SSE gradient in K_d490_, with higher values occurring in the western and north Mediterranean (Fig. 1B). On the other hand, surface PAR show higher values in the south-eastern part of the basin (Fig. 1C). Similar patterns have been already documented in the Mediterranean Sea from in situ bio-optical observations^75^ and ocean colour data^36,76–78^. As a result, higher values of K_dPAR_ generally occur in the north and western part of the basin (Fig. 1D). The highest irradiances at seabed, in terms of both irradiance (Fig. 1E) and percentage of surface PAR (Fig. 1F), are present in the Adriatic Sea and in the south-central Mediterranean Sea whilst the central and south-eastern Mediterranean Sea show the largest portion of seafloor with over 50% of surface PAR. From our analysis, the Adriatic Sea, which is the smaller sub-region (139,300 km^2^), has over 50% of seafloor under mesophotic conditions (71,889 km^2^, Fig. 3B). On the contrary, the Western Mediterranean Sea is the most extended sub-region but shows a portion of seabed under mesophotic conditions (86,656 km^2^, 10.3%) lower than those of the Ionian Sea, of the central Mediterranean and of the Aegean-Levantine Sea (126,003 km^2^ and 92,901 km^2^, 16.3% and 12.27%, respectively, Fig. 3B).

### Light and depth values of the Mediterranean mesophotic zone

**Figure 4:**
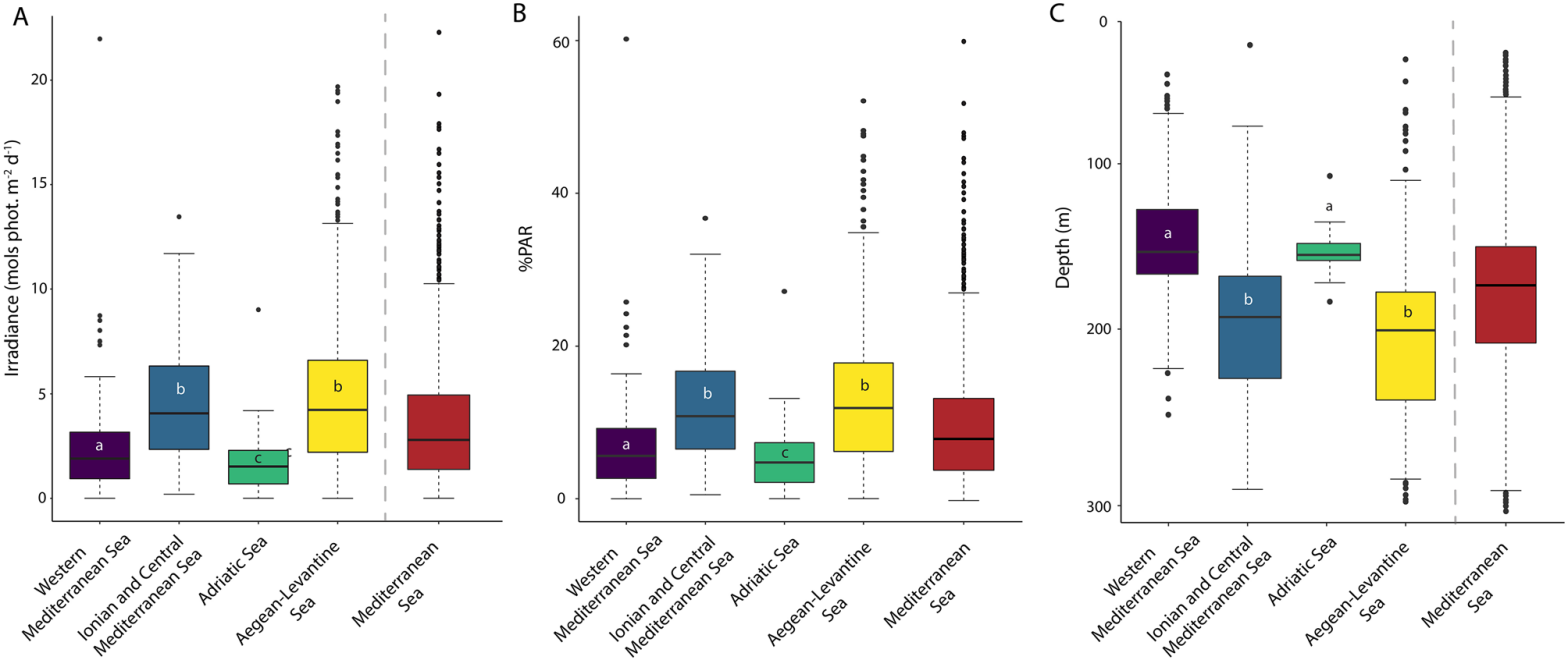
Depth and light ranges of the estimated mesophotic zone. Boxplots showing the irradiance (mol photons m^−2^ day^−1^) (**A**) and the percentage of PAR light (**B**) at the upper mesophotic limit (i.e., 30 m water depth) for the different Mediterranean sub-regions; (**C**) reports the depth corresponding to the lower mesophotic limit (i.e., at 0.0001 mol photons m^−2^ day^−1^ irradiance) for the Mediterranean sub-regions. The red boxes represent the values for the entire basin. Letters refer to statistically significant differences obtained with Kruskal–Wallis and Dunn’s multiple comparison tests (Table 1). Within each box the horizontal line represents median value. Boxes extend from the 25th to the 75th percentile. The vertical lines indicate the most extreme values within 1.5 interquartile range of the 25th and 75th percentile.

The irradiance and percentage of surface PAR at 30 m depth (i.e., upper limit of the mesophotic zone) reveal differences among the Mediterranean sub-regions in line with the NNW-SSE gradients shown by K_d490_, K_dPAR_ and the quantity of PAR light at seabed (Fig. 4A,B). The Aegean-Levantine Sea shows the highest values of irradiance at the upper border, with an average value of 4.97 ± 0.18 mol photons m^−2^ day^−1^ (13.3 ± 0.5% surface PAR), followed by the Ionian and Central Mediterranean Sea (4.69 ± 0.18 mol photons m^−2^ day^−1^, 12.2 ± 0.5%), the Western Mediterranean Sea (2.21 ± 0.10 mol photons m^−2^ day^−1^, 6.4 ± 0.3%) and the Adriatic Sea (1.61 ± 0.09 mol photons m^−2^ day^−1^, 5.0 ± 0.3%). At the large basin scale, the irradiance at 30 m depth is 3.69 ± 0.09 mol photons m^−2^ day^−1^, corresponding to 9.8 ± 0.2% surface PAR.

The Kruskal–Wallis test (p < 0.01) and the pairwise comparison by the Dunn’s multiple comparison test revealed significant differences in the irradiance and percentage of surface PAR at the upper limit of the mesophotic zone among the Mediterranean sub-regions (Fig. 4A,B, Table 1). In particular, the Ionian and Central Mediterranean Sea and the Aegean-Levantine Sea sub-regions are statistically different from the Western Mediterranean Sea and the Adriatic Sea.

**Table 1:** Results of Kruskal–Wallis test and the post hoc Dunn's multiple comparisons test, showing significance of differences in the irradiance and percent of surface PAR at the upper border, and in the depth of the lower mesophotic border among Mediterranean subregions.

Kruskal–Wallis test	χ^2^	df	pValue	
Irradiance at upper border (30 m)	292.4	3	< 0.001**	
% surface PAR at upper border	508.06	3	< 0.001**	
Depth at lower border (0.0001 mol phot. m^−2^ day^−1^)	249.57	3	< 0.001**	

Dunn's multiple comparison test	Subregions	ADR	ALS	ICMED
Irradiance at upper border (30 m)	WMED	0.004*	< 0.001**	< 0.001**
	ICMED	< 0.001**	0.99	
	ALS	< 0.001**		
% surface PAR at upper border	WMED	0.03*	< 0.001**	< 0.001**
	ICMED	< 0.001**	0.97	
	ALS	< 0.001**		
Depth at lower border (0.0001 mol phot. m^−2^ day^−1^)	WMED	0.98	< 0.001**	< 0.001**
	ICMED	< 0.001**	0.7575	
	ALS	< 0.001**		

*WMED* Western Mediterranean Sea, *ADR* Adriatic Sea, *ICMED* Ionian Sea and central Mediterranean Sea, *ALS* Aegean-Levantine Sea.

p value significance level; *p ≤ 0.05; **p ≤ 0.001.

The depth of the lower limit of the mesophotic zone, defined at 0.0001 mol photons m^−2^ day^−1^ (i.e., ca. 0.0005% of surface PAR) shows values in line with the north-west–south-east gradient observed for K_d490_, surface PAR and K_dPAR_ (Fig. 4C). The mean depth for the Western Mediterranean Sea and the Adriatic Sea is 152 ± 1.3 m and 154 ± 1.2 m, respectively. The penetration of light along the water column increases towards the south-eastern part of the basin, with the Aegean-Levantine Sea and the Ionian and Central Mediterranean Sea displaying values of 198 ± 1.9 m and 190 ± 2.5 m, respectively (Fig. 4C). The Kruskal–Wallis and post-hoc pairwise tests indicate that the differences between the north-west (Western Mediterranean Sea and Adriatic Sea) and the south-east (Ionian Sea and Central Mediterranean Sea and Aegean Levantine Sea) sub-regions are statistically significant (Fig. 4C, Table 1). The mean value for the entire Mediterranean Sea is 175.5 ± 1.2 m.

### Literature data

**Figure 5:**
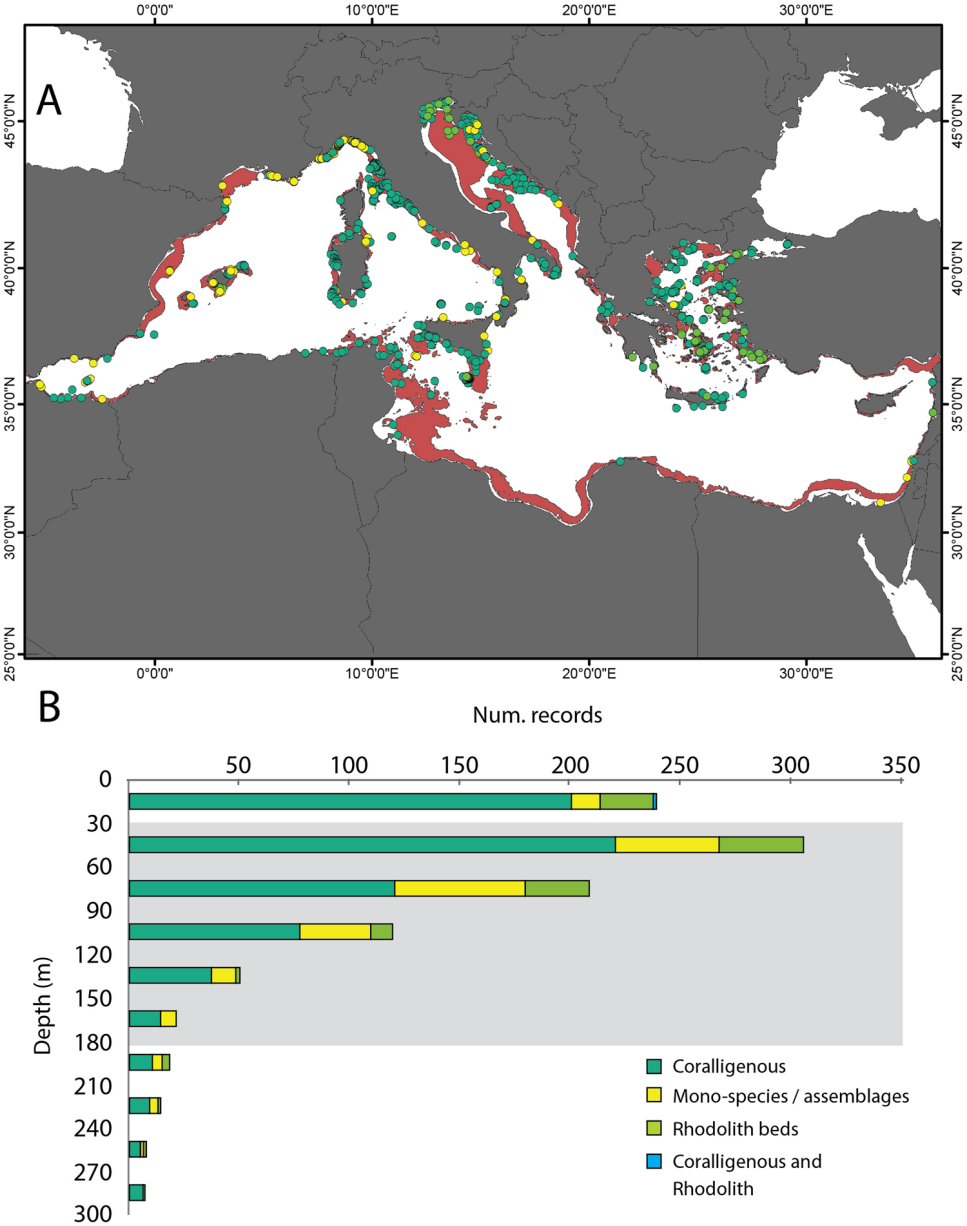
Spatial information on Mediterranean mesophotic ecosystems in the literature. (**A**) Map showing the locations of mesophotic assemblages sourced from literature data and the sites with coralligenous formations and rhodolith beds from the MEDISEH database. The red shaded area indicates the portions of seabed characterized by mesophotic conditions (from this study). Map generated using ArcMap 10.5 software (© ESRI, https://desktop.arcgis.com). (**B**) Depth range of the different benthic mesophotic assemblages. The grey shaded area marks the depth range predicted as under mesophotic conditions in the Mediterranean Sea (30–175.5 m).

Most of the literature on Mediterranean mesophotic ecosystems focused on the northern part of the basin, with limited observations from the southern region (Fig. 5A). In particular, the Aegean Sea and the Italian and Croatian margins present the highest number of observations, followed by studies along the French and Spanish coasts (Fig. 5A). Out of 997 occurrences of mesophotic assemblages extracted from literature, 685 observations (69%) occur in seabed characterized by mesophotic conditions, based on criteria adopted in the present study (Fig. 5A,B). The remaining ca. 24.7% of the dataset (246 records) reports observations above 30 m depth and 6.6% (66 records) below the lower limit of the mesophotic conditions (i.e., < 0.0001 mol photons m^−2^ day^−1^, Fig. 5B).

## Discussion

The mesophotic zone in the Mediterranean Sea hosts a variety of different biological components, from coralligenous formations to rhodolith beds, sponge grounds, gorgonian forests and oyster reefs, depending on bathymetry, geographic location in the basin and local factors^32,33,45,46,49–53,55^. These ecosystems supply essential goods and services, acting as nursery and spawning grounds for fishes, potential carbon sinks, and represent a unique natural heritage^71,73,79,80^. Providing a spatial definition of the mesophotic zone is, therefore, of primary importance to support the implementation of conservation policies addressing such ecosystems and inform management plans^14^.

Although the bathymetric range associated with the mesophotic zone in the Mediterranean differs in the literature^31^, most of the studies so far have adopted the depth range defined for tropical and subtropical latitudes, between 30 and 150 m^13^. The lower limit of the mesophotic zone at temperate latitudes is, however, strictly related to the penetration of light in the water column that vary largely with geographic location. The gradient of irradiance not only defines the border of the mesophotic zone but also concurs to shape the structure of mesophotic assemblages, along with other physical and biochemical factors^30,63,81^, controlling the presence of obligate phototrophic organisms and ultimately defining the bathymetric range of the mesophotic zone.

Our analysis of the irradiance at the upper limit of the mesophotic zone (30 m depth) for the entire Mediterranean Sea (3.69 ± 0.09 mol photons m^−2^ day^−1^, i.e., 9.8 ± 0.2% of surface PAR), and the presence of live crustose coralline algae at irradiance level down to 0.0001 mol photons m^−2^ day^−1^ (i.e., 0.0005%^60,64^) suggest that mesophotic conditions occur at irradiance between ca. 4 and 0.0001 mol photons m^−2^ day^−1^, corresponding to surface PAR levels between ca. 10 and 0.0005%. This range agrees with observations on coralligenous and rhodolith bed assemblages, which are commonly found in dim light conditions where irradiance can be as low as 0.05% and 0.0005% of surface light, respectively^60,61^.

At the Mediterranean basin scale, the depth of the lower limit of the mesophotic zone is 175.5 ± 1.2 m, consistent with the bathymetric definition in previous studies^31^. The threshold of 0.0001 mol photons m^−2^ day^−1^ (0.0005% of surface PAR) as the lower limit for mesophotic conditions ensures to include portions of seabed characterized by low light conditions but excludes areas below 200 m depth, commonly referred as the beginning of the “deep-sea” (below 200 m depth^82,83^), thus avoiding an overlap between different bathymetric domains.

The comparison between our estimation and the location of literature records on mesophotic ecosystems showed that 69% of literature records on mesophotic ecosystems fell within the proposed area (Fig. 3B), lending support to the reliability of the results.

Using our approach, more than 377,000 km^2^ of the seafloor, corresponding to ca. 15% of the entire Mediterranean Sea surface area, is under mesophotic conditions. The attenuation of solar radiation increases with increasing depth and is affected by the amount of dissolved and particulate matter suspended in the water column, which is correlated with primary productivity, hydrodynamic conditions, and terrigenous influences^84^. Freshwater inputs (e.g., rivers and precipitations) carry substantial quantities of terrigenous organic and mineral particles, dissolved organic matter, and release large amounts of nutrients to the sea. For instance, in the Western Mediterranean Sea, the estimated portion of seabed under mesophotic conditions is lower than in the eastern subregions, although covering a larger area. Intense algal blooms occur in the Western Mediterranean Sea in spring when the surface layer stabilizes, and less frequently in autumn when the thermocline is eroded^76^. These factors can increase the attenuation of light penetration in the water column, as confirmed by the higher values of K_dPAR_ in the north-western Mediterranean Sea.

The extension of seabed receiving sunlight and the amount of irradiance reaching the seabed have also been proved to be influenced by the morphology of the seafloor^30,81^. For example, some sectors of the Western Mediterranean Sea are characterized by vertical or highly steep bottom whose extent is not properly represented on a horizontal projection. Consequently, the portion of seabed under mesophotic conditions appears restricted. This, nevertheless, does not necessarily mean that mesophotic ecosystems are not present in such situations but rather that they might distribute along highly steep bottoms. Several mesophotic communities have been, indeed, documented colonizing rocky vertical walls in the Western Mediterranean (e.g.,^42,45,85^). On the contrary, the large (more than 50%) area predicted as under mesophotic conditions in the Adriatic Sea does not imply more abundant mesophotic communities, but it is likely related to the geomorphology of the subregion. The Adriatic Sea is, in fact, characterized by gentle slopes, presenting an average bottom depth of about 35 m that increases moving to the south (140 m on average, central Adriatic), with the two Pomo/Jabuka Depressions reaching 260 m^86^. Estimating the spatial extent of the mesophotic zone using the penetration of light in the water column can only identify areas with mesophotic light regime. A complex combination of variables concurs in determining the presence of mesophotic communities, such as biogeochemical factors and type of substrate along with biotic and stochastic processes. Consequently, the extent of seabed under mesophotic conditions does not necessarily represent the probability of finding mesophotic communities.

Our results, however, suggest that the quantity of light at seabed calculated from satellite K_d490_ concentration is a valid tool to spatially constrain the mesophotic zone, helping in the identification of areas suitable for mesophotic ecosystems, and their related biodiversity and ecological services. Although the depth range 30–150 m has been proved to be associated with mesophotic conditions at tropical latitudes, the greatest strength of using a physical approach based on satellite data is that it guarantees to appreciate variations in the bathymetric extension of the mesophotic zone related to local factors and seasonal variability, especially when dealing with non-tropical situations. Estimating the penetration of light along the water column assures also to identify differences in the depth of occurrence of obligate phototrophic organisms with geographic position, ultimately providing information on the structure of mesophotic assemblages and the ecological functions they provide.

By including information on the light regime, our approach can also estimate the spatial and bathymetric extension of the mesophotic zone in situations characterized by data deficiency. For instance, the southern Mediterranean would appear devoid of mesophotic assemblages from an analysis of the literature due to the few accounts available to date. Our method, instead, highlighted that large portions of this sector of the Mediterranean Sea might be under mesophotic conditions, enabling their inclusion in the quantitative assessment of the mesophotic zone at the basin scale.

Direct measurements of irradiances and turbidity along depth would surely represent the best method to validate the penetration of light obtained from satellite data. Our results, nevertheless, show that literature records can be successfully used as groundtruthing surrogate when on-field irradiance measurements are not available, providing a degree of confidence associated to the spatial definition of the mesophotic zone. The method presented is not meant to be a “one-size-fits-all” solution but an approach to estimate the depth range associated with mesophotic conditions based solely on satellite data. This is particularly important to furnish robust information to be included in management plans and to orient future explorations. In this context, by comparing the distribution of areas estimated as presenting mesophotic conditions with information on local and regional anthropogenic impacts, our methodology might help in identifying areas potentially hosting mesophotic communities threatened by human activities.

Finally, an approach that integrates information on the light regime will also permit to explore the variations in the spatial and vertical extension of the mesophotic zone in response to future climatic conditions of the oceans. The IPCC prediction foresees that the vertical extension of the photic zone might vary in the near future as a consequence of the decline of nutrient supply, marine productivity and phytoplankton community, leading to an increase in water transparency and, thus, light penetration^65,69^. Irradiances associated with the mesophotic zone might occur deeper than at present by the end of the century, forcing a physiological response in benthic mesophotic species and surely influencing the structure, distribution of mesophotic ecosystems. By drawing its current extension, our estimation represents a baseline for studying how the depth range of the mesophotic zone in the Mediterranean Sea will vary in relation to future conditions of the oceans, permitting the identification of areas most affected by climate changes.

## Materials and methods

### Quantity of PAR at seabed

The quantity of light at seabed was calculated following the methodology proposed by^87^ that derives the diffuse attenuation coefficient for PAR light (K_dPAR_) as a function of the diffuse attenuation coefficient for light at 490 nm (K_d490_) according to the following formula:

1 $${\text{K}}_{{{\text{dPAR}}}} = 0.0{665} + 0.{874}*{\text{K}}_{{{\text{d49}}0}} - 0.00{121}*{\text{K}}_{{{\text{d49}}0}}{^{{ - {1}}}}$$

The intensity of light (E_z_) at a given depth (z) is then calculated from the intensity of light entering the ocean (E_0_) and K_dPAR_ by applying the Beer’s Law^35^:

2 $${\text{E}}_{{\text{z}}} = {\text{E}}_{0} {\text{e}}^{ - {\text {K}_{\text {dPAR}}\text z}}$$

The diffuse attenuation coefficients K_d490_ and K_dPAR_ are also influenced by the directionality of the light field, but this dependence has proven to be weak at mesophotic depths^35,74^. We assumed K_d490_ as a good representation of the optical water quality for the purposes of our study.

Daily data on K_d490_ in m^−1^ were used to calculate the 17-years mean for the Mediterranean Sea for the period 2002–2018. The average PAR at surface for the same period was estimated from daily average values in mol photons m^−2^ day^−1^. The data were obtained from the open-access repository NASA Ocean Colour (https://oceancolor.gsfc.nasa.gov/), at a resolution of 4 km. Bathymetric data were downloaded from open-access repository EMODnet (https://www.emodnet-bathymetry.eu/) with a horizontal resolution of ca. 100 m. Data on K_d490_ and surface PAR were processed to match the resolution of the bathymetry using an upscaling approach that approximates conditions at the seafloor (sensu^88^) and that has been demonstrated to work effectively in different scales and situations (e.g.,^89^). The quantity of PAR reaching the seabed was, then, calculated following the equations described above. Finally, the percentage of PAR at seabed was estimated as the ratio between light at seabed (E_z_) and surface PAR in each pixel, multiplied by 100. The processing was performed in R software using the packages “raster”, “sp” and “rgdal”^90^. The estimated irradiance at seabed is available at https://doi.org/10.5281/zenodo.5863103.

### Bathymetric definition of the mesophotic zone in the Mediterranean Sea

The upper limit of the mesophotic zone in the Mediterranean Sea was set at 30 m, according to literature evidence constraining zooxanthellate corals mainly above this water depth (e.g.,^32^). The portions of seabed above 30 m depth were then excluded from the subsequent analysis.

The lower limit of the mesophotic zone was identified based on the minimum value of PAR that is known to support photosynthetic metabolism. In^60^ and more recently^64^, live crustose coralline algae have been observed at PAR level down to 0.0001 daily photon dose (mol phot m^−2^ day^−1^) corresponding to 0.0005%, which is the lowest value recorded so far. This value was used as the lower limit for mesophotic conditions. The seabed with estimated irradiance at seabed lower than 0.0001 mol photons m^−2^ day^−1^ was excluded. The remaining dataset was converted into a polygon using the “Raster to Polygon” tool ArcGIS 10.5.

Setting the limits at 30 m depth and at the minimum daily photon dose documented as sustaining net positive photosynthesis, however, omits information on light conditions at the upper and on depth at the lower. Consequently, we generated a point every 20 km along both borders and extracted the corresponding irradiance, percent PAR and depth values to provide ranges of light (average ± standard error) and depth for the mesophotic zone in the Mediterranean Sea. Their variation across the basin was tested by dividing the dataset into subsets corresponding to the sub-regions outlined by the European Union (EU) Marine Strategy Framework Directive (2008/56/CE): Western Mediterranean Sea, Adriatic Sea, Ionian Sea and Central Mediterranean Sea and the Aegean-Levantine Sea (Figs. 2, 3).

### Spatial distribution of benthic mesophotic communities

We compiled a list of 112 bibliographical records on benthic mesophotic ecosystems in the Mediterranean Sea, through an extensive bibliographic search in ISI Web of Science, Scopus, and the mesophotic.org website (http://www.mesophotic.org/). A first screening aimed at removing studies not related to benthic ecosystems (e.g., targeting fish fauna), then we selected only those reporting the exact location of the investigated assemblage. Out of 112 documents, 69 fulfilled the criteria. We integrated this information with the distribution of coralligenous and rhodolith bed habitats from the MEDISEH database based on literature reviews^57^. Overall, 1407 relevant locations to our study were selected for the Mediterranean basin. Among these, 38 records wrongly located on land and 317 falling outside the extent of satellite data (e.g., too close to coastline) were removed. Finally, 55 sites deeper than 500 m were not considered, representing potential outliers. The final dataset consisted in 997 locations of mesophotic assemblages covering the entire Mediterranean Sea. The complete list of the literature records and bibliography search methodology can be found at https://doi.org/10.5281/zenodo.5645586.

Records were classified depending on the principal target in 4 different groups: “Generic assemblages” include mono-species and community records, “Coralligenous” groups information on coralligenous habitats, “Rhodolith beds” for occurrences of rhodolith beds and “Coralligenous and rhodolith” includes both coralligneous and rhodolith-beds records. The dataset was georeferenced and the depth and the quantity of light at seabed were extracted for each location using the package ‘raster’ in R software.

### Statistical analysis

The depth, irradiances and PAR percentage at seabed did not meet the assumptions of normality and homoscedasticity verified by using the Shapiro–Wilk test and Levene test, respectively (package “car”, R software;^90^). Therefore, we performed nonparametric Kruskal–Wallis tests followed by post hoc pairwise comparisons using a Dunn’s multiple comparison test with Bonferroni adjusted p-values to analyze the differences among sub-regions of the Mediterranean Sea.

## Data Availability

All data are available at https://doi.org/10.5281/zenodo.5645586 and https://doi.org/10.5281/zenodo.5863103.

## References

[1] Hoegh-Guldberg O, Bruno JF (2010). The impact of climate change on the World’s Marine Ecosystems. Science.

[2] Hewitt JE, Ellis JI, Thrush SF (2016). Multiple stressors, nonlinear effects and the implications of climate change impacts on marine coastal ecosystems. Glob. Change Biol..

[3] Sweetman AK, Thurber AR, Smith CR, Levin LA, Mora C, Wei C-L, Gooday AJ, Jones DOB, Rex M, Yasuhara M, Ingels J, Ruhl HA, Frieder CA, Danovaro R, Würzberg L, Baco A, Grupe BM, Pasulka A, Meyer KS, Dunlop KM, Henry L-A, Roberts JM (2017). Major impacts of climate change on deep-sea benthic ecosystems. Elementa Sci. Anthropocene..

[4] Leslie HM (2005). A synthesis of marine conservation planning approaches. Conserv. Biol..

[5] Oppel S, Bolton M, Carneiro APB, Dias MP, Green JA, Masello JF, Phillips RA, Owen E, Quillfeldt P, Beard A, Bertrand S, Blackburn J, Boersma PD, Borges A, Broderick AC, Catry P, Cleasby I, Clingham E, Creuwels J, Crofts S, Cuthbert RJ, Dallmeijer H, Davies D, Davies R, Dilley BJ, Dinis HA, Dossa J, Dunn MJ, Efe MA, Fayet AL, Figueiredo L, Frederico AP, Gjerdrum C, Godley BJ, Granadeiro JP, Guilford T, Hamer KC, Hazin C, Hedd A, Henry L, Hernández-Montero M, Hinke J, Kokubun N, Leat E, Tranquilla LM, Metzger B, Militão T, Montrond G, Mullié W, Padget O, Pearmain EJ, Pollet IL, Pütz K, Quintana F, Ratcliffe N, Ronconi RA, Ryan PG, Saldanha S, Shoji A, Sim J, Small C, Soanes L, Takahashi A, Trathan P, Trivelpiece W, Veen J, Wakefield E, Weber N, Weber S, Zango L, Daunt F, Ito M, Harris MP, Newell MA, Wanless S, González-Solís J, Croxall J (2018). Spatial scales of marine conservation management for breeding seabirds. Mar. Policy.

[6] Manea E, Bianchelli S, Fanelli E, Danovaro R, Gissi E (2020). Towards an ecosystem-based marine spatial planning in the deep Mediterranean Sea. Sci. Total Environ..

[7] Aylesworth L, Phoonsawat R, Suvanachai P, Vincent ACJ (2017). Generating spatial data for marine conservation and management. Biodivers. Conserv..

[8] Lesser MP, Slattery M, Leichter JJ (2009). Ecology of mesophotic coral reefs. J. Exp. Mar. Biol. Ecol..

[9] James NP, Ginsburg RN, Ginsburg RN (1979). The Seaward Margin of Belize Barrier and Atoll Reefs: Morphology, Sedimentology, Organism Distribution, and Late Quaternary History.

[10] Ginsburg RN, Harris PM, Eberli GP, Swart PK (1991). The growth potential of a bypass margin, Great Bahama Bank. J. Sediment. Res..

[11] Pyle RL, Copus JM, Loya Y, Puglise KA, Bridge TCL (2019). Mesophotic coral ecosystems: Introduction and overview. Mesophotic Coral Ecosystems. Coral Reefs of the World.

[12] Kahng SE, Garcia-Sais JR, Spalding HL, Brokovich E, Wagner D, Weil E, Hinderstein L, Toonen RJ (2010). Community ecology of mesophotic coral reef ecosystems. Coral Reefs.

[13] Hinderstein LM, Marr JCA, Martinez FA, Dowgiallo MJ, Puglise KA, Pyle RL, Zawada DG, Appeldoorn R (2010). Theme section on “Mesophotic coral ecosystems: Characterization, ecology, and management”. Coral Reefs.

[14] J. A. Turner, D. A. Andradi-Brown, A. Gori, P. Bongaerts, H. L. Burdett, C. Ferrier-Pagès, C. R. Voolstra, D. K. Weinstein, T. C. L. Bridge, F. Costantini, E. Gress, J. Laverick, Y. Loya, G. Goodbody-Gringley, S. Rossi, M. L. Taylor, N. Viladrich, J. D. Voss, J. Williams, L. C. Woodall, G. Eyal. in *Mesophotic Coral Ecosystems*, *Coral Reefs of the World*, 989–1003 (Y. Loya, K. A. Puglise, T. C. L. Bridge, Eds). (Springer International Publishing, 2019). 10.1007/978-3-319-92735-0_52.

[15] Baker EK, Puglise KA, Harris PT, United Nations Environment Programme, GRID-Arendal (2016). Mesophotic Coral Ecosystems: A Lifeboat for Coral Reefs?.

[16] Lang JC (1974). Biological Zonation at the Base of a Reef: Observations from the submersible Nekton Gamma have led to surprising revelations about the deep fore-reef and island slope at Discovery Bay, Jamaica. Am. Scientist..

[17] J. K. Reed. Deepest distribution of Atlantic hermatypic corals discovered in the Bahamas. in *Proceedings of the 5th International Coral Reef Symposium* (1985), Vol. 6, 249–254.

[18] Hanisak MD, Blair SM (1988). The deep-water macroalgal community of the East Florida continental shelf (USA). Helgolander Meeresunters..

[19] Aponte NE, Ballantine DL (2001). Depth distribution of algal species on the deep insular fore reef at Lee Stocking Island, Bahamas. Deep Sea Res. Part I.

[20] Fricke HW, Vareschi E, Schlichter D (1987). Photoecology of the coral *Leptoseris fragilis* in the Red Sea twilight zone (an experimental study by submersible). Oecologia.

[21] Kahng S, Maragos J (2006). The deepest, zooxanthellate scleractinian corals in the world?. Coral Reefs.

[22] Maragos JE, Jokiel PL (1986). Reef corals of Johnston Atoll: One of the world’s most isolated reefs. Coral Reefs.

[23] Bridge TCL (2011). Variability in mesophotic coral reef communities along the Great Barrier Reef, Australia. Mar. Ecol. Progress Series.

[24] Lesser MP, Slattery M (2011). Phase shift to algal dominated communities at mesophotic depths associated with lionfish (*Pterois volitans*) invasion on a Bahamian coral reef. Biol. Invasions.

[25] Slattery M, Lesser MP, Loya Y, Puglise KA, Bridge TCL (2019). The Bahamas and Cayman Islands. Mesophotic Coral Ecosystems.

[26] Slattery M, Lesser MP, Brazeau D, Stokes MD, Leichter JJ (2011). Connectivity and stability of mesophotic coral reefs. J. Exp. Mar. Biol. Ecol..

[27] Lesser MP, Slattery M, Laverick JH, Macartney KJ, Bridge TC (2019). Global community breaks at 60 m on mesophotic coral reefs. Glob. Ecol. Biogeogr..

[28] Tamir R, Eyal G, Kramer N, Laverick JH, Loya Y (2019). Light environment drives the shallow-to-mesophotic coral community transition. Ecosphere.

[29] Laverick JH, Green TK, Burdett HL, Newton J, Rogers AD (2019). Depth alone is an inappropriate proxy for physiological change in the mesophotic coral Agaricia lamarcki. J. Mar. Biol. Assoc. UK.

[30] Lesser MP, Mobley CD, Hedley JD, Slattery M (2021). Incident light on mesophotic corals is constrained by reef topography and colony morphology. Mar. Ecol. Prog. Ser..

[31] Cerrano C, Bastari A, Calcinai B, Camillo CD, Pica D, Puce S, Valisano L, Torsani F (2019). Temperate mesophotic ecosystems: Gaps and perspectives of an emerging conservation challenge for the Mediterranean Sea. Eur. Zool. J..

[32] Idan T (2018). Shedding light on an East-Mediterranean mesophotic sponge ground community and the regional sponge fauna. Mediterr. Mar. Sci..

[33] Idan T, Goren L, Shefer S, Brickner I, Ilan M (2020). Does depth matter? Reproduction pattern plasticity in two common sponge species found in both mesophotic and shallow waters. Front. Mar. Sci..

[34] Enrichetti F, Dominguez-Carrió C, Toma M, Bavestrello G, Betti F, Canese S, Bo M (2019). Megabenthic communities of the Ligurian deep continental shelf and shelf break (NW Mediterranean Sea). PLoS ONE.

[35] Kahng SE, Akkaynak D, Shlesinger T, Hochberg EJ, Wiedenmann J, Tamir R, Tchernov D, Loya Y, Puglise KA, Bridge TCL (2019). Coral reefs of the world. Mesophotic Coral Ecosystems.

[36] D’Ortenzio F, Ribera d’Alcalà M (2009). On the trophic regimes of the Mediterranean Sea: A satellite analysis. Biogeosciences.

[37] Christaki U, Van Wambeke F, Lefevre D, Lagaria A, Prieur L, Pujo-Pay M, Grattepanche J-D, Colombet J, Psarra S, Dolan JR, Sime-Ngando T, Conan P, Weinbauer MG, Moutin T (2011). Microbial food webs and metabolic state across oligotrophic waters of the Mediterranean Sea during summer. Biogeosciences.

[38] Rossi V, Ser-Giacomi E, López C, Hernández-García E (2014). Hydrodynamic provinces and oceanic connectivity from a transport network help designing marine reserves. Geophys. Res. Lett..

[39] Basterretxea G, Font-Muñoz JS, Salgado-Hernanz PM, Arrieta J, Hernández-Carrasco I (2018). Patterns of chlorophyll interannual variability in Mediterranean biogeographical regions. Remote Sens. Environ..

[40] Tanhua T (2013). Repeat hydrography in the Mediterranean Sea, data from the Meteor cruise 84/3 in 2011. Earth Syst. Sci. Data.

[41] Bethoux JP (1979). Budgets of the Mediterranean Sea-their dependance on the local climate and on the characteristics of the Atlantic waters. Oceanol. Acta.

[42] Azov Y (1991). Eastern Mediterranean—A marine desert?. Mar. Pollut. Bull..

[43] Pinardi N, Zavatarelli M, Arneri E, Crise A, Ravaioli M (2006). The physical, sedimentary and ecological structure and variability of shelf areas in the Mediterranean Sea. The Sea.

[44] Rodolfo-Metalpa R, Montagna P, Aliani S, Borghini M, Canese S, Hall-Spencer JM, Foggo A, Milazzo M, Taviani M, Houlbrèque F (2015). Calcification is not the Achilles’ heel of cold-water corals in an acidifying ocean. Glob. Change Biol..

[45] Bo M, Bava S, Canese S, Angiolillo M, Cattaneo-Vietti R, Bavestrello G (2014). Fishing impact on deep Mediterranean rocky habitats as revealed by ROV investigation. Biol. Cons..

[46] Cau A, Follesa MC, Moccia D, Alvito A, Bo M, Angiolillo M, Canese S, Paliaga EM, Orrù PE, Sacco F, Cannas R (2015). Deepwater corals biodiversity along roche du large ecosystems with different habitat complexity along the south Sardinia continental margin (CW Mediterranean Sea). Mar. Biol..

[47] L. Bramanti, M. C. Benedetti, R. Cupido, S. Cocito, C. Priori, F. Erra, M. Iannelli, G. Santangelo. in *Marine Animal Forests: The Ecology of Benthic Biodiversity Hotspots*, 529–548 (S. Rossi, L. Bramanti, A. Gori, C. Orejas Eds.) (Springer International Publishing, 2017). 10.1007/978-3-319-21012-4_13.

[48] Capdevila P, Linares C, Aspillaga E, Riera JL, Hereu B (2018). Effective dispersal and density-dependence in mesophotic macroalgal forests: Insights from the Mediterranean species *Cystoseira zosteroides*. PLoS ONE.

[49] Angeletti L, Canese S, Cardone F, Castellan G, Foglini F, Taviani M (2020). A brachiopod biotope associated with rocky bottoms at the shelf break in the central Mediterranean Sea: Geobiological traits and conservation aspects. Aquat. Conserv. Mar. Freshwat. Ecosyst..

[50] Angeletti L, Taviani M (2020). Offshore *Neopycnodonte* Oyster Reefs in the Mediterranean Sea. Diversity.

[51] Castellan G, Angeletti L, Taviani M, Montagna P (2019). The yellow coral *Dendrophyllia cornigera* in a warming ocean. Front. Mar. Sci..

[52] Corriero G, Pierri C, Mercurio M, Marzano CN, Tarantini SO, Gravina MF, Lisco S, Moretti M, Giosa FD, Valenzano E, Giangrande A, Mastrodonato M, Longo C, Cardone F (2019). A Mediterranean mesophotic coral reef built by non-symbiotic scleractinians. Sci. Rep..

[53] Chimienti G (2020). Vulnerable Forests of the Pink Sea Fan *Eunicella verrucosa* in the Mediterranean Sea. Diversity.

[54] Gori A, Rossi S, Bramanti L, Gori A, Orejas C (2017). Animal forests in deep coastal bottoms and continental shelf of the Mediterranean Sea. Marine Animal Forests: The Ecology of Benthic Biodiversity Hotspots.

[55] Goren L, Idan T, Shefer S, Ilan M (2021). Macrofauna inhabiting massive demosponges from shallow and mesophotic habitats along the Israeli Mediterranean Coast. Front. Mar. Sci..

[56] Santín A (2018). Sponge assemblages on the deep Mediterranean continental shelf and slope (Menorca Channel, Western Mediterranean Sea). Deep Sea Res. Part I.

[57] Martin CS, Giannoulaki M, De Leo F, Scardi M, Salomidi M, Knittweis L, Pace ML, Garofalo G, Gristina M, Ballesteros E, Bavestrello G, Belluscio A, Cebrian E, Gerakaris V, Pergent G, Pergent-Martini C, Schembri PJ, Terribile K, Rizzo L, Ben Souissi J, Bonacorsi M, Guarnieri G, Krzelj M, Macic V, Punzo E, Valavanis V, Fraschetti S (2014). Coralligenous and maërl habitats: predictive modelling to identify their spatial distributions across the Mediterranean Sea. Sci. Rep..

[58] D. Basso, L. Babbini, A. A. Ramos-Esplá, M. Salomidi. in *Rhodolith/Maërl Beds: A Global Perspective*, *Coastal Research Library*, 281–298 (R. Riosmena-Rodríguez, W. Nelson, J. Aguirre, Eds.) (Springer International Publishing, 2017). 10.1007/978-3-319-29315-8_11.

[59] Foster MM, Amado Filho GM, Kamenos NA, Riosmena-Rodríguez R, Steller DL (2013). Rhodoliths and rhodolith beds. Res. Discoveries Revolut. Sci. Through Scuba..

[60] Littler MM, Littler DS, Dennis Hanisak M (1991). Deep-water rhodolith distribution, productivity, and growth history at sites of formation and subsequent degradation. J. Exp. Mar. Biol. Ecol..

[61] Ballesteros E (2006). Mediterranean coralligenous assemblages: a synthesis of present knowledge. Oceanogr. Mar. Biol. Annu. Rev.

[62] Smith TB, Blondeau J, Nemeth RS, Pittman SJ, Calnan JM, Kadison E, Gass J (2010). Benthic structure and cryptic mortality in a Caribbean mesophotic coral reef bank system, the Hind Bank Marine Conservation District, U. S. Virgin Islands. Coral Reefs.

[63] Markager S, Sand-Jensen K (1992). Light requirements and depth zonation of marine macroalgae. Mar. Ecol. Prog. Ser..

[64] Runcie JW, Gurgel CFD, Mcdermid KJ (2008). In situ photosynthetic rates of tropical marine macroalgae at their lower depth limit. Eur. J. Phycol..

[65] Bindoff, N. L., *et al.* Chapter 5: Changing ocean, marine ecosystems, and dependent communities. Intergovernmental panel of climate change. in *IPCC Special Report on the Ocean and Cryosphere in a Changing Climate* 447–587 (2019).

[66] Tweedley JR, Warwick RM, Potter IC, Hughes RN, Hughes DJ, Smith IP, Dale AC (2016). The contrasting ecology of temperate macrotidal and microtidal estuaries. Oceanography and Marine Biology: An Annual Review.

[67] Arias-Ortiz A, Serrano O, Masqué P, Lavery PS, Mueller U, Kendrick GA, Rozaimi M, Esteban A, Fourqurean JW, Marbà N, Mateo MA, Murray K, Rule MJ, Duarte CM (2018). A marine heatwave drives massive losses from the world’s largest seagrass carbon stocks. Nat. Clim. Change..

[68] Chen N, Krom MD, Wu Y, Yu D, Hong H (2018). Storm induced estuarine turbidity maxima and controls on nutrient fluxes across river-estuary-coast continuum. Sci. Total Environ..

[69] Agusti S, Lubián LM, Moreno-Ostos E, Estrada M, Duarte CM (2019). Projected changes in photosynthetic picoplankton in a warmer subtropical ocean. Front. Mar. Sci..

[70] Lesser MP, Slattery M (2020). Will coral reef sponges be winners in the Anthropocene?. Glob. Change Biol..

[71] Ponti M, Turicchia E, Ferro F, Cerrano C, Abbiati M (2018). The understorey of gorgonian forests in mesophotic temperate reefs. Aquat. Conserv. Mar. Freshwat. Ecosyst..

[72] Enrichetti F, Bo M, Morri C, Montefalcone M, Toma M, Bavestrello G, Tunesi L, Canese S, Giusti M, Salvati E, Bertolotto RM, Bianchi CN (2019). Assessing the environmental status of temperate mesophotic reefs: A new, integrated methodological approach. Ecol. Ind..

[73] Soares MO, Tavares TCL, Carneiro PBM (2019). Mesophotic ecosystems: Distribution, impacts and conservation in the South Atlantic. Diversity Distributions..

[74] Mobley CD, Mobley CD (1994). Light and Water: Radiative Transfer in Natural Waters.

[75] Marty J-C, Chiavérini J (2002). Seasonal and interannual variations in phytoplankton production at DYFAMED time-series station, northwestern Mediterranean Sea. Deep Sea Res. Part II.

[76] Morel A, André J-M (1991). Pigment distribution and primary production in the western Mediterranean as derived and modeled from coastal zone color scanner observations. J. Geophys. Res. Oceans..

[77] Antoine D, Morel A, André J-M (1995). Algal pigment distribution and primary production in the eastern Mediterranean as derived from coastal zone color scanner observations. J. Geophys. Res. Oceans..

[78] Mayot N, D’Ortenzio F, Ribera d’Alcalà M, Lavigne H, Claustre H (2016). Interannual variability of the Mediterranean trophic regimes from ocean color satellites. Biogeosciences.

[79] S. Kahng, J. M. Copus, D. Wagner. in *Marine Animal Forests: The Ecology of Benthic Biodiversity Hotspots*, 185–206 (S. Rossi, L. Bramanti, A. Gori, C. Orejas, Eds.) (Springer International Publishing, 2017). 10.1007/978-3-319-21012-4_4.

[80] Chimienti G (2021). Effects of global warming on Mediterranean coral forests. Sci. Rep..

[81] Lesser MP, Slattery M, Mobley CD (2021). Incident light and morphology determine coral productivity along a shallow to mesophotic depth gradient. Ecol. Evol..

[82] Spalding MD, Fox HE, Allen GR, Davidson N, Ferdaña ZA, Finlayson M, Halpern BS, Jorge MA, Lombana A, Lourie SA, Martin KD, McManus E, Molnar J, Recchia CA, Robertson J (2007). Marine ecoregions of the world: A bioregionalization of coastal and shelf areas. Bioscience.

[83] Danovaro R, Fanelli E, Canals M, Ciuffardi T, Fabri M-C, Taviani M, Argyrou M, Azzurro E, Bianchelli S, Cantafaro A, Carugati L, Corinaldesi C, Haan WP, Dell’Anno A, Evans J, Foglini F, Galil B, Gianni M, Goren M, Greco S, Grimalt J, Güell-Bujons Q, Jadaud A, Knittweis L, Lopez JL, Sanchez-Vidal A, Schembri PJ, Snelgrove P, Vaz S, Angeletti L, Barsanti M, Borg JA, Bosso M, Brind’Amour A, Castellan G, Conte F, Delbono I, Galgani F, Morgana G, Prato S, Schirone A, Soldevila E (2020). Towards a marine strategy for the deep Mediterranean Sea: Analysis of current ecological status. Mar. Policy..

[84] Saulquin B, Hamdi A, Gohin F, Populus J, Mangin A, d’Andon OF (2013). Estimation of the diffuse attenuation coefficient KdPAR using MERIS and application to seabed habitat mapping. Remote Sens. Environ..

[85] Grinyó J, Garriga A, Soler-Membrives A, Santín A, Ambroso S, López-González PJ, Díaz D (2020). Soft corals assemblages in deep environments of the Menorca Channel (Western Mediterranean Sea). Progress Oceanogr..

[86] Artegiani A, Paschini E, Russo A, Bregant D, Raicich F, Pinardi N (1997). The Adriatic Sea general circulation. Part I: Air–sea interactions and water mass structure. J. Phys. Oceanogr..

[87] Morel A, Huot Y, Gentili B, Werdell PJ, Hooker SB, Franz BA (2007). Examining the consistency of products derived from various ocean color sensors in open ocean (Case 1) waters in the perspective of a multi-sensor approach. Remote Sens. Environ..

[88] Davies AJ, Guinotte JM (2011). Global habitat suitability for framework-forming cold-water corals. PLoS ONE.

[89] Georgian SE, Kramer K, Saunders M, Shedd W, Roberts H, Lewis C, Fisher C, Cordes E (2020). Habitat suitability modelling to predict the spatial distribution of cold-water coral communities affected by the Deepwater Horizon oil spill. J. Biogeogr..

[90] R. C. Team, *R: A language and environment for statistical computing (3. 5. 1)[Computer software]. R Foundation for Statistical Computing* (2020).

